# Lectin microarray profiling and monosaccharide analysis of bovine milk immunoglobulin G oligosaccharides during the first 10 days of lactation

**DOI:** 10.1002/fsn3.950

**Published:** 2019-04-02

**Authors:** Shane Feeney, Jared Q. Gerlach, Helen Slattery, Michelle Kilcoyne, Rita M. Hickey, Lokesh Joshi

**Affiliations:** ^1^ Teagasc Food Research Centre Moorepark Fermoy, Co. Cork Ireland; ^2^ Glycoscience Group National Centre for Biomedical Engineering Science National University of Ireland Galway Galway Ireland; ^3^ Carbohydrate Signalling Group Discipline of Microbiology School of Natural Sciences National University of Ireland Galway Galway Ireland

**Keywords:** glycosylation, immunoglobulin, lactation, lectin, microarray, milk

## Abstract

Immunoglobulin G (IgG) in bovine milk is credited with ensuring efficient passive immunity for newborn calves. Bovine milk IgG glycosylation may also have positive impacts on the health of nonbovine consumers of cow's milk. Milk IgG's glycosylation contributes to effector function and may also protect it from protease digestion, allowing IgG to reach the intestine for absorption. However, relatively little is known about changes in milk IgG oligosaccharide presentation and composition over early lactation. In this work, IgG was isolated from milk pooled from three cows at four time points over the first 10 days of lactation postparturition. Purified IgG was labeled with a fluorescent dye and interrogated with a microarray consisting of 48 carbohydrate‐binding proteins (lectins) from plant, fungal, and bacterial sources. Lectin microarray profiles suggested that only subtle changes in the glycosylation of IgG occurred during days 2 and 3 of lactation, but by day 10, the lectin profile diverged from the other three time points. Monosaccharide analysis carried out after hydrolysis confirmed that the ratios of oligosaccharide components remained relatively stable through day 3 and also that sialylation was substantially reduced by day 10. The differences that were observed for glycosylation suggest that different functionalities associated with IgG glycosylation may be required in the first days of life.

## 1 INTRODUCTION

Milk‐derived immunoglobulin G (IgG) has key roles in the mammalian immune system. IgG found in bovine colostrum and milk and consists of two identical 50 kDa, class γ heavy chains, and two identical 25 kDa light chains linked together by disulfide bonds that are arranged in a tetrameric quaternary structure. The concentration of IgG in bovine milk is known to be highest in colostrum during the first 5 days postpartum (Takimori et al., 2011) and contributes to passive immunity in the calf. The transfer of IgG from mother to calf is critically important for triggering an innate immune response in the Ig‐poor newborn calves to prevent pathogenic infection (El‐Fattah, Rabo, El‐Dieb, & El‐Kashef, 2012). It is possible that the glycan structures present on IgG may be critical factors in the initiation of this innate immune response (Marth & Grewal, 2008). At the same time, milk IgG transfer is believed to promote the colonization of the calf's gut by beneficial bacteria. The alterations in the bovine milk N‐glycome, specifically that of IgG over lactation, may be an evolutionary adaptation to aid the changing biological requirements of the calf's immune system and its ever‐developing gut microbiota.

Studies have shown that bovine IgG can assist in the prevention of human enteric diseases caused by rotavirus, *Escherichia coli, Cryptosporidium* spp., and *Shigella flexneri* (Hurley & Theil, 2011). In humans, dietary IgG uptake in humans occurs when its Fc portions bind to FcRn in the intestinal lumen where it is assimilated by the *lamina propria* which triggers a local mucosal immune response that augments immune surveillance and defense of the mucosal lining. Glycosylation changes of major human milk components including IgG, lactoferrin (hLF), and bovine milk fat globule membrane (MFGM) have been observed over the course of lactation (Barboza et al., 2012; Bondt et al., 2014; Lauc et al., 2013; O'Riordan, Kane, Joshi, and Hickey, 2014; Wilson et al., 2008). The alteration in total milk protein glycosylation throughout lactation has been largely attributed to qualitative and quantitative N‐linked glycosylation changes of IgG, which is the main immunoglobulin found in colostrum (Takimori et al., 2011).

Bovine IgG oligosaccharides have been reported to contain fucose (Fuc), galactose (Gal), and mannose (Man) structures in addition to sialic acids (*N*‐glycolylneuraminic acid (Neu5Gc) and *N*‐acetylneuraminic acid (Neu5Ac)) and variations in the glycosylation of total bovine milk glycoproteins (with an emphasis on IgG) during early lactation (day 1 and weeks 1–4) have recently been reported (Takimori et al., 2011). Bovine colostrum IgG has a particularly high sialic acid content with milk samples from day 1 postpartum reported to have the highest Neu5Gc:Neu5Ac ratio, with a gradual decline in Neu5Gc concentration observed at later time points (Takimori et al., 2011). IgG α‐(2→6)‐linked sialylation is associated with decreased inflammation following Fc receptor binding (Marth & Grewal, 2008). Sialic acid on other milk glycoproteins is thought to play important roles in binding *E. coli* and *Salmonella enteritidis* (Feeney, Ryan, Kilcoyne, Joshi, & Hickey, 2017; Nakajima et al., 2005), promoting the growth of the bifidobacteria strains such as *Bifidobacterium breve*,* B. bifidum,* and *B. infantis* (Idota, Kawakami, & Nakajima, 1994), preventing *Helicobacter pylori* colonization (Wang et al., 2007), inhibiting haemagglutination by the influenza virus (Kawasaki et al., 1993), and promoting calcium‐binding properties (Jaques, Brown, Barrett, Brey, & Weltner, 1977).

The current study focuses on IgG glycosylation over the first 10 days postpartum since it is during this period that IgG is most abundant and critical for newborn immunity. Soluble IgG was isolated from milk samples pooled from three cows at four time points using a scaled‐down, optimized affinity liquid chromatography‐based method. Lectin microarrays were used to profile the temporal glycosylation changes of IgG which was accompanied by chromatographic monosaccharide analysis.

## 2 MATERIALS AND METHODS

### 2.1 Materials

The Sartobind^®^ S SingleSep nano 1 ml capsules were from Sartorius Stedim Biotech (Göttingen, Germany). Protein G columns (5 ml, catalogue no. 17‐0405‐01) were from GE Healthcare Bio‐Sciences AB (UK). Nexterion^®^ Slide H microarray slides were supplied by Schott AG (Mainz, Germany). 8‐well gasket slide and incubation cassette system were from Agilent Technologies Ireland, Ltd., (Cork, Ireland). Pure, unlabeled lectins were purchased from EY Laboratories, Inc. (San Mateo, CA, USA), Vector Laboratories, Ltd. (Orton Southgate, UK), or Sigma‐Aldrich Co. (Dublin, Ireland). Carboxylic acid succinimidyl ester Alexa Fluor^®^ 647 (AF647) fluorescent label was from Life Technologies (Carlsbad, CA, USA). Vivaspin^®^ 5 Centrifugal Ultra Filter columns (5 ml, 25 kDa molecular weight cutoff (MWCO)) were acquired from Thermo Fisher Scientific (Dublin, Ireland). Amicon Ultracel 100 kDa MWCO centrifugal ultrafiltration units were supplied by Millipore, Cork, Ireland. Precast 4%–12% Bis‐Tris polyacrylamide gels, sodium lauryl dodecyl sulfate (LDS) sample buffer, MOPS running buffer and antioxidant (all NuPAGE^®^ branded), and also Invitrogen SimplyBlue^™^ SafeStain and PageRuler^™^ Prestained Protein Ladder were sourced from Thermo Fisher, Dublin. Monosaccharide standards (99% purity or greater) L‐fucose (Fuc), D‐galactose (Gal), D‐glucose (Glc), *N*‐acetyl‐D‐glucosamine (GlcNAc), *N*‐acetyl‐D‐galactosamine (GalNAc), D‐mannose (Man)*,* D‐glucosamine (GlcN), D‐galactosamine (GalN), Neu5Ac and Neu5Gc were supplied by Sigma‐Aldrich Co. (Dublin, Ireland). Bradford reagent and IgG standard were supplied by Sigma‐Aldrich Co. All other reagents were supplied by Sigma‐Aldrich Co. and were of the highest grade available.

### 2.2 Sample collection

Morning milk was collected daily from three Holstein–Friesian cows at Teagasc Research Centre, Moorepark, Fermoy, Co. Cork, Ireland. Samples were collected from each animal at days 1, 2, 3, and 10 postparturition and were pooled at the same time point. Whole milk samples were defatted using an FT15 disk bowl centrifuge (Armfield Ltd., Ringwood, UK) which separated the cream from the milk. Samples were lyophilized and stored in a desiccator at 4°C until further use.

### 2.3 IgG isolation

Soluble IgG was isolated from pooled skimmed bovine milk at four time points over the first 10 days of lactation using a 5 ml Protein G column using conditions similar to the manufacturer's instructions. In brief, bovine skimmed milk powder was suspended in 20 mm phosphate buffer (pH 7.0) to give a concentration of 10 mg/ml which was loaded on to the column at 5 ml/min. The column was washed with 20 mm phosphate buffer for 10 column volumes (CV), and then the bound IgG was eluted with 5 CV of 100 mm glycine‐HCl (pH 2.7) at 5 ml/min. The recovered IgG fraction was neutralized with 1 m Tris‐HCl (pH 7.6). The IgG fractions were concentrated and buffer exchanged into distilled water using a 100 kDa MWCO centrifugal ultrafiltration unit. IgG samples were then lyophilized to dryness and stored at 4°C until further use.

### 2.4 Size exclusion chromatography and SDS‐PAGE analysis

The purity of IgG samples was assessed using size exclusion high‐performance liquid chromatography (SE‐HPLC) and SDS‐PAGE as previously described (O'Riordan, Kane, et al., 2014; O'Riordan, Gerlach, et al., 2014). In brief, SE‐HPLC was performed using a TSK‐Gel column (G3000SW, Tosoh Biosciences LLC, Tokyo, Japan) eluted with 25 mm NaH_2_PO_4_ and 100 mm Na_2_SO_4_, pH 7.0.

Immunoglobulin G samples were electrophoresed using precast 4%–12% Bis‐Tris gels. All samples were diluted 1:10 with LDS sample buffer, and 5 μg IgG was loaded into each well. A molecular mass ladder and an IgG standard were run alongside the purified IgG samples. Gels were resolved at 200 V constant and variable current for 50 min using MOPS buffer in the inner chamber and MOPS buffer containing 0.25% antioxidant was used in the outer chamber. Protein bands were visualized within the gels using Coomassie‐based SafeStain following the manufacturer's procedure.

### 2.5 Fluorescent labeling of IgG and glycoproteins

Immunoglobulin G samples and the bovine asialofetuin (ASF) standard were labeled with AF647 (λ_ex_ 650 nm, λ_em_ 665 nm) in 100 mm sodium bicarbonate, pH 8.3, in the dark as previously described (Gerlach, Kilcoyne, & Joshi, 2014). Briefly, 1 mg of AF647 was dissolved in 100 μl DMSO, and 10 μl of the dissolved dye was added to 500 μl of IgG sample (2 mg/ml in 100 mm sodium bicarbonate, pH 8.3) and incubated for 1 hr in the dark at room temperature. Labeled IgG was then separated from unconjugated dye in phosphate‐buffered saline (PBS; 10 mm sodium phosphate, 137 mm NaCl, 2.7 mm KCl, 2 mm KH_2_PO_4_, pH 7.4) using a 3 kDa MWCO centrifugal filter. Labeled IgG samples were quantified for protein content and substitution according to manufacturer's instructions. Samples were stored in the dark at 4°C until further use.

### 2.6 Construction and incubation of lectin microarrays

A panel of 48 lectins was prepared at a concentration of 0.5 mg/ml in PBS, pH 7.4, supplemented with 1 mm of the appropriate haptenic sugar (Table S1). Lectins were printed at approximately 1 nl per feature on Nexterion^®^ Slide H microarray slides using a sciFLEXARRAYER S3 piezoelectric printer (Scienion AG, Berlin, Germany) as previously described (Gerlach et al., 2014). The lectins were maintained at 10°C in a 62% relative humidity environment during printing. Each microarray slide contained eight replicate subarrays, with each lectin spotted in replicates of six per subarray. To ensure complete conjugation, these slides were then incubated in a humidity chamber overnight at room temperature. Residual functional groups were deactivated by incubation in 100 mm ethanolamine in 50 mm sodium borate, pH 8.0, for 1 hr at room temperature. Each slide was washed with PBS, pH 7.4, containing 0.05% Tween^®^‐20 (PBS‐T) three times for 3 min per wash, once with PBS, centrifuged dry (450 × *g*, 5 min) and stored at 4°C with desiccant until use.

Prior to use, the lectin microarray slides were allowed to equilibrate to room temperature for 30 min under desiccant. Microarrays and fluorescent IgGs were protected from light throughout each experiment. Fluorescently labeled IgG samples and the ASF control were diluted to 0.5 μg/ml in Tris‐buffered saline supplemented with Ca^2+^ and Mg^2+^ ions (TBS; 20 mm Tris‐HCl, 100 mm NaCl, 1 mm CaCl_2_, 1 mm MgCl_2_) pH 7.2 with 0.05% Tween^®^‐20 (TBS‐T). 70 μl of each diluted sample (0.5 and 1.0 μg/ml for sets 1 and 2, respectively) was applied to each well of the microarray and incubated in the dark (1 hr, 23°C, 4 rpm) as previously described (Gerlach et al., 2014; O'Riordan, Kane, et al., 2014; O'Riordan, Gerlach, et al., 2014). Following incubation, microarrays were washed twice in TBS‐T and once in TBS for 3 min each wash. Finally, microarrays were dried by centrifugation and imaged in an Agilent G2505B (Agilent Technologies) microarray scanner using the Cy5 channel (633 nm excitation, 80% PMT, 5 μm/pixel resolution).

### 2.7 Data extraction

Microarray data extraction was performed as previously described (Gerlach et al., 2014; Kilcoyne, Gerlach, Kane, & Joshi, 2012). In brief, GenePix Pro v6.1.0.4 (Molecular Devices, Berkshire, UK) was used to extract raw intensity values from image files using a proprietary *.gal file which enabled the identification of 230 μm printed lectin spots using adaptive diameter (70%–130%) circular alignment. The data were then exported as text to Excel (version 2010, Microsoft). Local background‐corrected median feature intensity data (F633median‐B633) values were selected, and the median of six replicate spots per subarray was handled as a single data point for graphical and statistical analysis. Binding intensity data are represented in bar charts as the mean intensity with single standard deviation of all like experimental replicates.

### 2.8 Monosaccharide analysis

One mg IgG from each time point was hydrolyzed in 0.1 m HCl at 80°C for 1 hr to release the sialic acids (Gallagher, Morris, & Dexter, 1985). Water and bovine fetuin were hydrolyzed for background subtractions and positive hydrolysis controls, respectively. Sialic acid content was quantified by high pH anion exchange chromatography with pulsed amperometric detection (HPAEC‐PAD) on an ICS3000 system fitted with an electrochemical detector (Dionex, Sunnyvale, CA, USA). Elution points and quantities were established by running Neu5Ac and Neu5Gc standards under the following conditions. Briefly, 25 μl of each reconstituted sample was injected onto a CarboPac PA20 column (3 × 150 mm, Dionex) fitted with an amino trap column (3 × 30 mm) at a flow rate of 0.35 ml/min. Eluent A was 100 mm NaOH, and eluent B was 100 mm NaOH with 500 mm sodium acetate. Separation of Neu5Ac and Neu5Gc was achieved by gradient elution (0–10 min, 14%–60% eluent B) and finally a wash step at 60% eluent B for 1 min. Samples were injected in triplicate, and Neu5Ac and Neu5Gc were quantified referencing the standard curve. The column was re‐equilibrated at 14% eluent B for 6 min.

Neutral and *N*‐acetylated sugars were hydrolyzed and analyzed following a previously described method (Kilcoyne, Gerlach, Gough et al., 2012; Kilcoyne, Gerlach, Kane, et al., 2012) with minor modification. Briefly, 1 mg of each IgG sample was hydrolyzed by 2 N trifluoroacetic acid (TFA) at 100°C for 4 hr. Hydrolyzate was dried from water three times by lyophilization and stored dry until analyzed. Released sugars were dissolved in 50 μl of 18.2 MΩ water and subjected to HPAEC‐PAD. The equivalent of 0.3–2.0 μg of hydrolyzate was injected in triplicate. Isocratic elution (18 mm NaOH at 0.35 ml/min) from a CarboPac PA20 analytical column (3 × 150 mm) equipped with an amino trap column (3 × 30 mm) was performed over 18.5 min. The column was washed with 100 mm NaOH for 8 min and re‐equilibrated at 18 mm NaOH for 12 min between injections. The monosaccharide content of each sample was quantified from HPAEC‐PAD standard curves of Fuc, Gal, Man, Glc, GalN, and GlcN eluted under the same conditions, and the average quantity of each monosaccharide was calculated.

## 3 RESULTS

### 3.1 Purification of IgG from bovine milk

**Figure 1:**
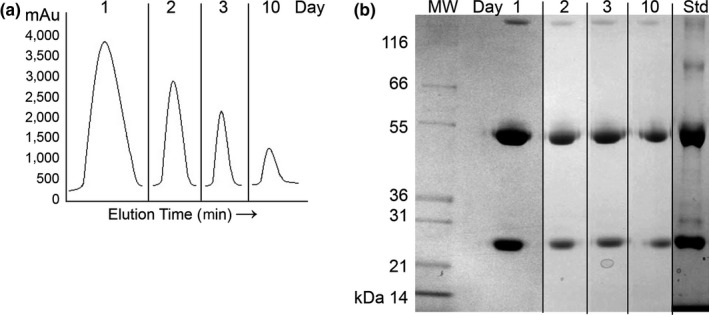
Purification of IgG from bovine milk. (a) Composite comparison of peaks from individual elution chromatograms showing the relative decrease in quantity of IgG obtained from equal loading of milk from each day. (b) Composite image of Coomassie‐stained SDS‐PAGE gels showing that the purity of the isolated IgG fractions (days 1, 2, 3, and 10) against the purchased IgG standard (Std). Lane MW is the molecular weight standard

After pooling by day, similar to commercial milk processing procedures, IgG was isolated from the skimmed milk samples collected from three cows from days 1, 2, 3, and 10 postparturition. The average IgG yield was highest at day 1 (12 mg/ml) postpartum but steadily decreased by days 2 (7 mg/ml) and 3 (4 mg/ml) before decreasing further by day 10 (0.5 mg/ml) (Figure 1a and also Figure S1). The purity of each IgG fraction eluted from the Protein G column was observed to be very high by reducing SDS‐PAGE (Figure 1b) with bands at approximately 50 kDa (reduced heavy chain) and 25 kDa (reduced light chain).

### 3.2 Lectin microarray profiling of IgG glycosylation

**Figure 2:**
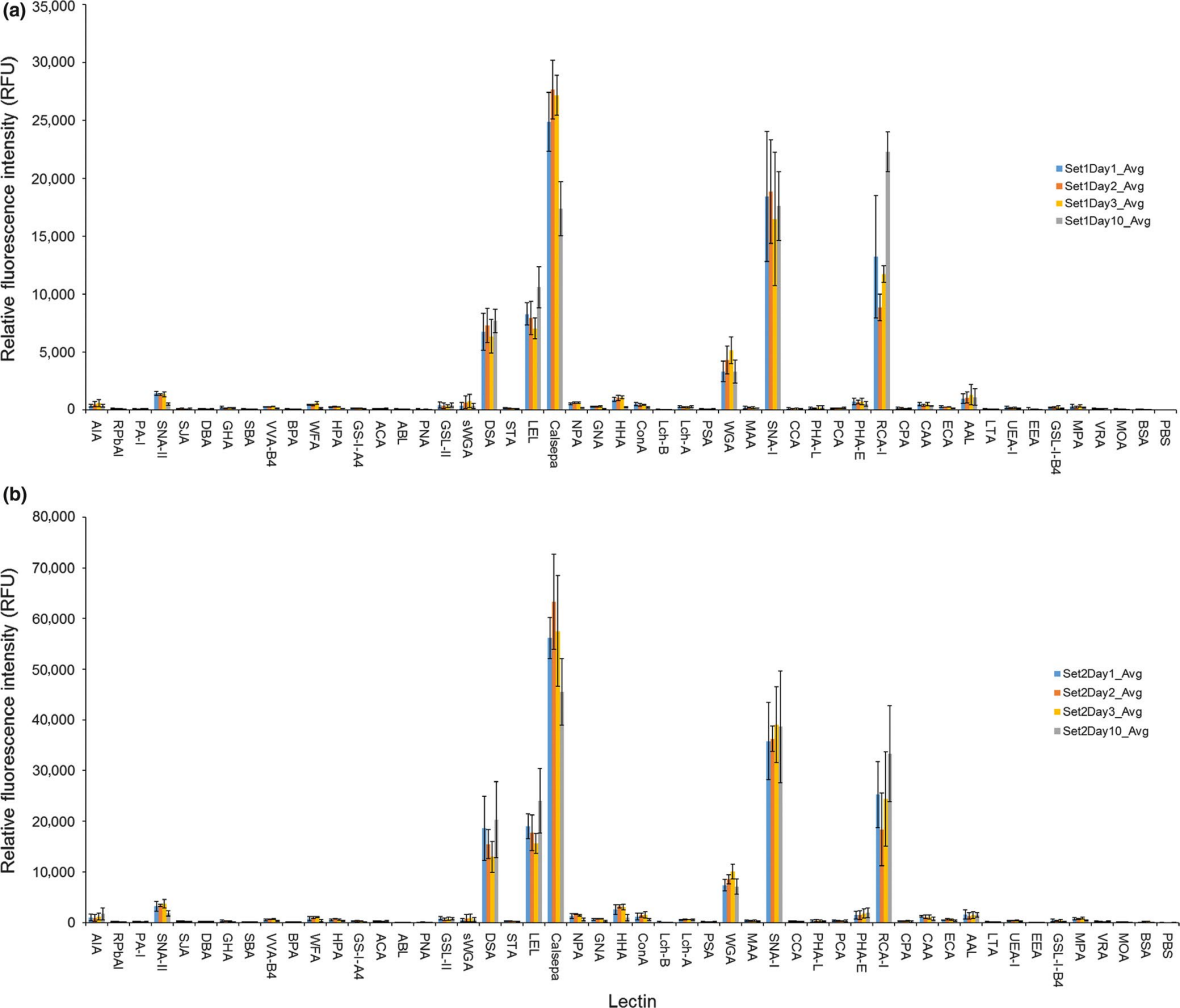
Comparison of the normalized profiles for IgG isolated from milk at days 1, 2, 3, and 10 postparturition. (a) Set one at 0.5 μg/ml loading and (b) set two at 1.0 μg/ml loading. Responses are averages for replicates with error bars representing ±1 *SD*

Following fluorescent labeling of the purified IgG samples from each time point, glycosylation was profiled by lectin microarray consisting of a panel of 48 immobilized lectins with previously established binding specificities (major carbohydrate structures reported to be preferential binding partners for each are included in Table S1). By titration, two concentrations were selected such that the response at each of the lectins was inside the dynamic range of the lectin microarray scanner (0–65,000 RFU, approx.). Each of the four fluorescently labeled samples was then profiled at the optimal concentrations in triplicate. Importantly, lectin microarray profiling performed at two different concentrations reinforced that the data were not generally influenced by loading concentration and that the results were highly reproducible (Figure 2a,b). A representative microarray image (Figure S2) is included in the Data S1.

**Figure 3:**
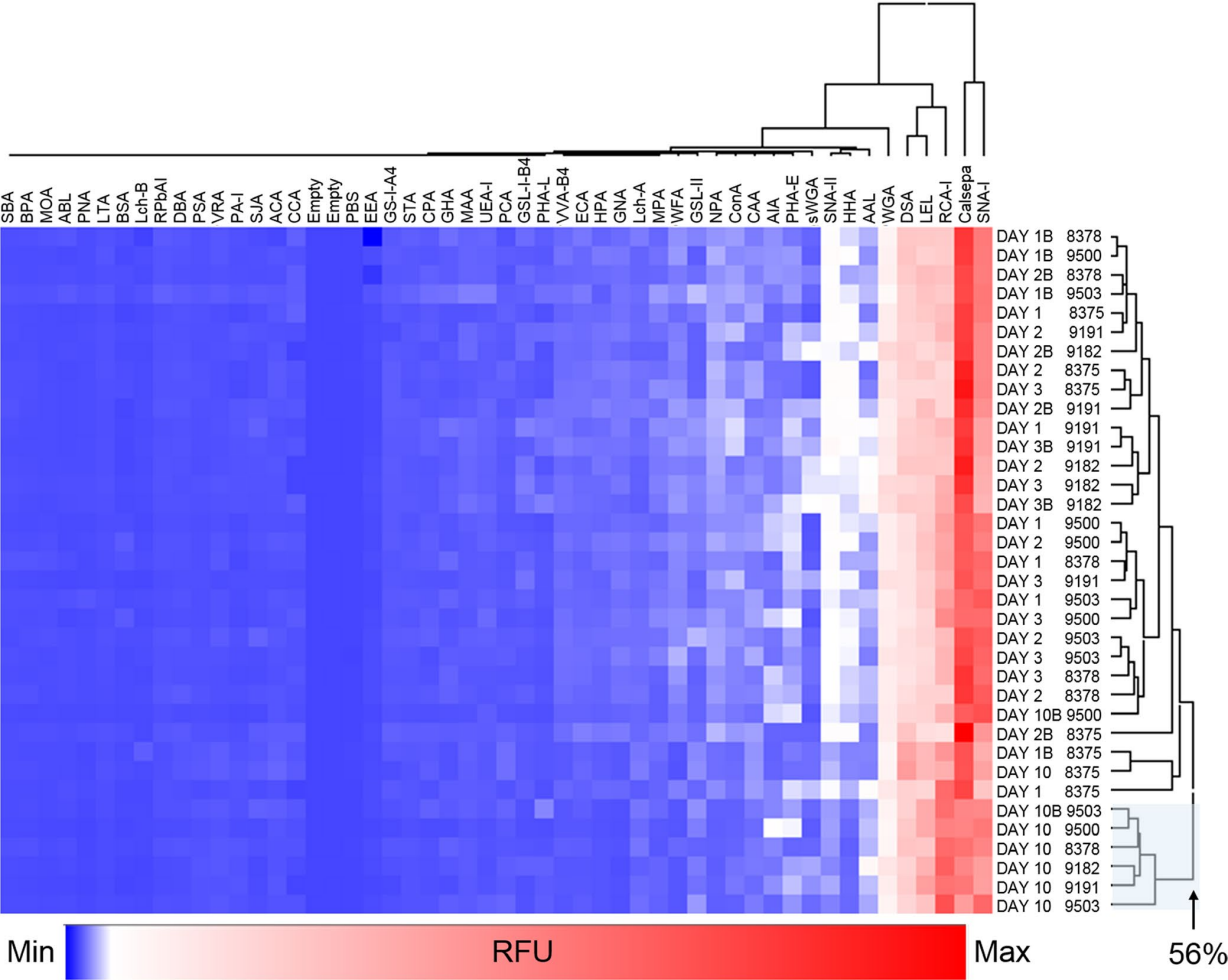
Unsupervised hierarchical clustering of the normalized lectin microarray responses for IgG from days 1, 2, 3, and 10 pooled milk samples (totaling 36 replicates, “B” indicates additional technical replicates of each sample compared on one single microarray). All data subjected to total intensity mean normalization prior to 2‐dimensional clustering using the average linkage, Euclidean distance method. Blue box at right indicates the cluster of day 10 profiles (6 out of 8 replicates) which demonstrated 56% minimum similarity to the balance of the samples with this clustering method. Day 1, *n* = 10; Day 2, *n* = 10; Day 3, *n* = 8; Day 10, *n* = 8). “Empty” represents the unprinted microarray slide surface, “PBS” buffer only, and “BSA” is bovine serum albumin

At both loading concentrations tested, the bovine IgG samples interacted with approximately 1/3 of the lectins comprising the microarray (Figure 2a,b). The most intense signals were observed for Calsepa, SNA‐I, LEL, DSA, WGA, AAL, and SNA‐II. While all samples displayed binding to these lectins, LEL and RCA‐I demonstrated greater affinity for day 10 IgG and Calsepa showed reduced intensity for the same sample. When all normalized replicate microarray data were compared by unsupervised hierarchical clustering analysis, the resulting groups further reinforced the observation that the only significant pattern differences for the bovine IgG samples occurred at day 10 (Figure 3). All but two of the day 10 IgG replicates clustered on a separate branch having 56% minimum similarity to all other replicates for all days from both sets of experiments (Figure 3, arrow).

### 3.3 Monosaccharide analysis

The monosaccharide composition of each IgG sample at each time point was assessed using HPAEC‐PAD which showed that IgG oligosaccharides are mainly composed of Man, Gal, GlcNAc, and Fuc (Table 1). Glc is not an expected component of mammalian N‐linked oligosaccharides on secreted proteins; however, Glc was observed at extremely low levels relative to Man and this was most likely introduced during the gel filtration steps used during the IgG purification or through airborne cellulose materials (e.g., paper fibers) during sample processing.

**Table 1:** Monosaccharide ratios calculated against moles of Man for each day's IgG sample

	Man	Gal	GlcNAc	Fuc	Glc	Neu5Ac	Neu5Gc
Day 1	1.00	0.59	0.37	0.32	0.09	0.28	0.10
Day 2	1.00	0.56	0.42	0.32	0.09	0.25	0.11
Day 3	1.00	0.55	0.44	0.34	0.03	0.21	0.12
Day 10	1.00	0.78	0.40	0.86	0.16	ND	0.03

Data derived from HPAEC‐PAD monosaccharide analysis of 1 mg IgG from each time point. ND: not detected.

Two forms of sialic acid, Neu5Ac and Neu5Gc, were detected in the IgG samples, with Neu5Ac being the most abundant form observed. Bovine IgG's relative sialic acid quantity was highest at day 1 and decreased until day 10 where sialic acid quantities were very low and only Neu5Gc could be reliably detected.

## 4 DISCUSSION

The lectin and monosaccharide analysis results in this study reflect previous reports which have shown bovine IgG to contain N‐linked oligosaccharides, most recently by Takimori et al. (2011). Changes in relative abundance and/or glycosylation of individual bovine milk glycoproteins over lactation have been previously demonstrated (O'Riordan, Kane, et al., 2014; O'Riordan, Gerlach, et al., 2014; Reinhardt & Lippolis, 2008; Ross et al., 2016; Takimori et al., 2011). The current IgG study correlates with the results observed by these authors. Cyclic variations in protein glycosylation have also been noted previously (O'Riordan, Kane, et al., 2014; O'Riordan, Gerlach, et al., 2014) and these may vary in phase and duration for individual animals. It is possible that the stable lectin profiles observed for IgG samples collected from early time points (specifically days 1, 2, and 3) occurred as a result of pooling the milk samples (i.e., the differences in profiles were smoothened as a result of the contributions of IgG with slightly different glycosylation for each animal). It would be interesting to test if these findings also vary between individual animals, however, as milk would be collected and pooled in an industrial setting, the pooled milk used during this study is more relevant to the vast majority of consumer milk products. Furthermore, lectin microarray analysis revealed that glycosylation of bovine IgG from pooled milk was different by day 10 of the milk maturation process and this was also reflected in the monosaccharide data.

All IgG samples bound to the Man‐binding lectins, GNA, and HHA, but with low intensity. Man residues are attached to the chitobiose (GlcNAc‐β‐(1→4)‐GlcNAc) core of N‐linked glycans and are more abundant in hybrid and high‐Man‐type N‐linked structures. On their own, the relatively low‐intensity binding to these lectins may indicate a low abundance of high‐Man‐ and hybrid‐type structures in these IgG samples or that complex‐type N‐linked glycosylation is favored. WGA and LEL both have an affinity for GlcNAc residues, while LEL has been reported to have greatest affinity for the chitobiose core of N‐linked structures. RCA‐I demonstrated high‐intensity binding of all IgG samples which may indicate the presence of terminal Gal residues in a type II *N*‐acetyllactosamine (LacNAc, Gal‐β‐(1→4)‐GlcNAc) linkage and this is similar to previous reports (Takimori et al., 2011). This observation is further supported by interaction with DSA, which has an affinity for GlcNAc residues, including those that are part of the type II LacNAc structure which comprises the termini of the antennae of nonsialylated complex‐ and hybrid‐type N‐linked oligosaccharides. High binding of all IgG samples to Calsepa, which favors binding to Man residues (Nakamura‐Tsuruta et al., 2008) but also has been suggested to be dependent upon bisecting GlcNAc residues (Nagae, Mishra, Hanashima, Tateno, & Yamaguchi, 2017), may corroborate DSA binding and additionally indicate the presence of accessible bisecting GlcNAc residues, often attributed to complex, biantennary glycans. Low‐intensity binding to PHA‐E and the lack of significant interaction with PHA‐L indicated that the N‐linked structures on the IgG samples were most likely biantennary and may also support the presence of bisecting GlcNAc.

All samples displayed low‐intensity binding at SNA‐II which suggested a low presence of terminal GalNAc and/or low presence of *N*,*N*′‐diacetyllactosamine (GalNAc‐β‐(1→4)‐GlcNAc, LacdiNAc). SNA‐II binding intensity was lowest for day 10 which was opposite of the increase in binding for RCA‐I observed for day 10 suggesting either an increased presence of type II LacNAc or simply the absence of sialylation on a consistent population of IgG oligosaccharides from mature milk. Interestingly, negligible levels of binding were observed for the samples toward ECA, which also binds to type II LacNAc oligomers.

WGA binding may indicate the presence of GlcNAc and/or sialic acid (Neu5Ac and/or Neu5Gc). Binding intensity with WGA increased over the first 3 days of lactation before returning to the initial lower intensity by day 10. The lectins SNA‐I and MAA have binding specificity for structures which include terminal α‐(2→6)‐ and α‐(2→3)‐linked sialic acids, respectively. IgG binding was only observed for SNA‐I, which suggested that the majority of sialic acid present in IgG was α‐(2→6)‐linked. This finding was in agreement with a previous report described for IgG where terminal α‐(2→6)‐linked sialic acid was further associated with the anti‐inflammatory activity of therapeutic human IgG (intravenous immunoglobulin) (Kaneko, Nimmerjahn, & Ravetch, 2006).

Monosaccharide distributions revealed by HPAEC‐PAD indicated that composition of the IgG oligosaccharides did vary slightly across time points, even for days 2 and 3 although the lectin microarray profiles for IgG collected on these days did not reflect significant pattern variation. At day 10, with respect to Man, the mole ratio of Gal edges upward slightly and there is also an increase in the relative amount of Fuc and a drop in sialylation. Curiously, the relative increase in Fuc suggested by the monosaccharide analysis was not reflected in the lectin microarray profiles for day 10.

It has previously been demonstrated that total sialylation decreases during the transition from bovine colostrum to mature milk and it has been suggested that sialic acid has an important role as part of IgG postpartum and may have immune or inflammatory effects (Takimori et al., 2011). This study shows that bovine IgG sialylation decreases subtly as lactation progresses across the first 3 days, but is markedly decreased by day 10. This decrease in sialic acid content could potentially be associated with a shift from di‐sialylated oligosaccharides in colostrum to mono‐sialylated glycans in later lactation. Sialic acid is important stabilizer of glycoproteins due to its calcium‐binding abilities which, in turn, affects glycoprotein antibacterial activities (Rossi et al., 2002). Bifidogenic activities have been attributed to Neu5Ac, so it may give IgG a prebiotic function (Idota et al., 1994). Similarly, developmental functions, including cognitive development and enhanced learning during early development in pig trials, have been associated with the consumption of protein‐bound sialic acids (Wang et al., 2007).

Neu5Ac was the most abundant form of sialic acid detected through HPAEC‐PAD analysis. Due to a mutation in the cytidine monophosphate‐*N*‐acetylneuraminic acid hydroxylase (*CMAH*) gene in the sialic acid pathway, humans are incapable of producing Neu5Gc. However, it has been discovered that Neu5Gc can be assimilated by human cells from the surrounding environment, including dietary sources such as milk products (Bardor, Nguyen, Diaz, & Varki, 2005; Padler‐Karavani et al., 2008). Free Neu5Gc and/or Neu5Gc glycoconjugates stimulate an immune response in mice that produces high titers of circulating antibodies (Hanganutziu–Deicher antibody) (Nguyen, Tangvoranuntakul, & Varki, 2005). Assimilation of Neu5Gc elicits an immune response in knockout mice and may elicit an immune response in humans (Padler‐Karavani et al., 2008). It may also have a role in metastasis and cancer development, as several carcinoma cell types have been shown to express Neu5Gc containing glycoconjugates (Malykh, Schauer, & Shaw, 2001). However, the relationship between dietary Neu5Gc and human health remains poorly understood. More research is needed to explore the potential health implications and bioactive functions of consuming the nonhuman, terminal residue Neu5Gc present on IgG oligosaccharides.

Currently, most products available from bovine colostrum milk are derived from unfractionated colostrum and not from its purified components such as IgG. The present study suggests that IgG could offer immense potential as a functional food ingredient by exploiting the temporal changes in IgG glycosylation as a source of novel bioactives pending further elucidation of the associated bioactivities.

## CONFLICT OF INTEREST

The authors of this work declare that they have no conflict of interest.

## ETHICS STATEMENT

This work was performed with milk samples noninvasively sourced from farm animals and fully conforms to Directive 2010/63/EU.
