# Supplementary material for: Lectin microarray profiling and monosaccharide analysis of bovine milk immunoglobulin G oligosaccharides during the first 10 days of lactation
Source: Food Sci Nutr. 2019 Apr 2;7(5):1564–72. doi: 10.1002/fsn3.950 (PMC6526632; doi:10.1002/fsn3.950)
Supplement: Supplementary file 1 [file FSN3-7-1564-s001.docx]

**Supplementary information**

**Lectin microarray profiling and monosaccharide analysis of bovine milk immunoglobulin G oligosaccharides during the first ten days of lactation**

Shane Feeney^1,2^, Jared Q. Gerlach^2^, Helen Slattery^1^, Michelle Kilcoyne^3^, Rita M. Hickey^1^ and Lokesh Joshi^2†^

^1^Teagasc Food Research Centre, Moorepark, Fermoy, Co. Cork, Ireland
^2^Glycoscience Group, National Centre for Biomedical Engineering Science, National University of Ireland Galway, Galway, Ireland

^3^Carbohydrate Signalling Group, Discipline of Microbiology, School of Natural Sciences, National University of Ireland Galway, Galway, Ireland.

^†^Corresponding author:
Lokesh Joshi

National University of Ireland Galway, Galway, Ireland.
Phone: +353 (0) 991495768
E-mail: lokesh.joshi@nuigalway.ie

**Table S1**

Lectins present on the microarray, their ligands and print sugars

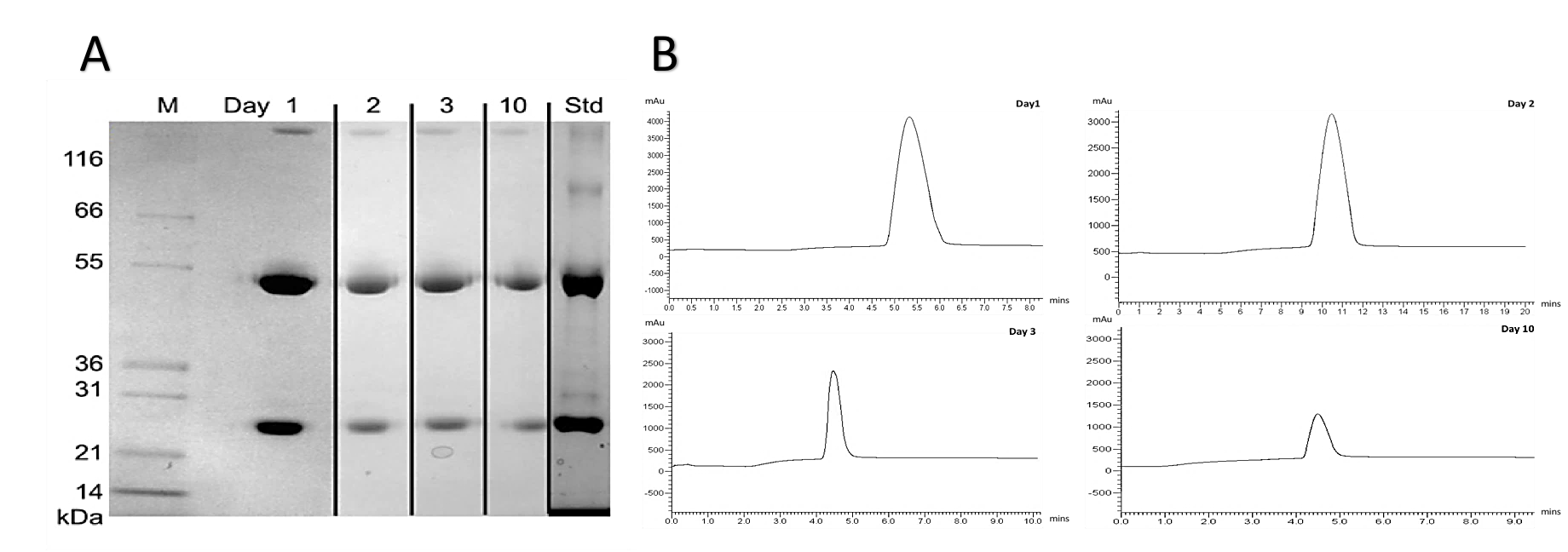


**Fig. S1.** Chromatograms detailing the elution of IgG for each day as indicated.


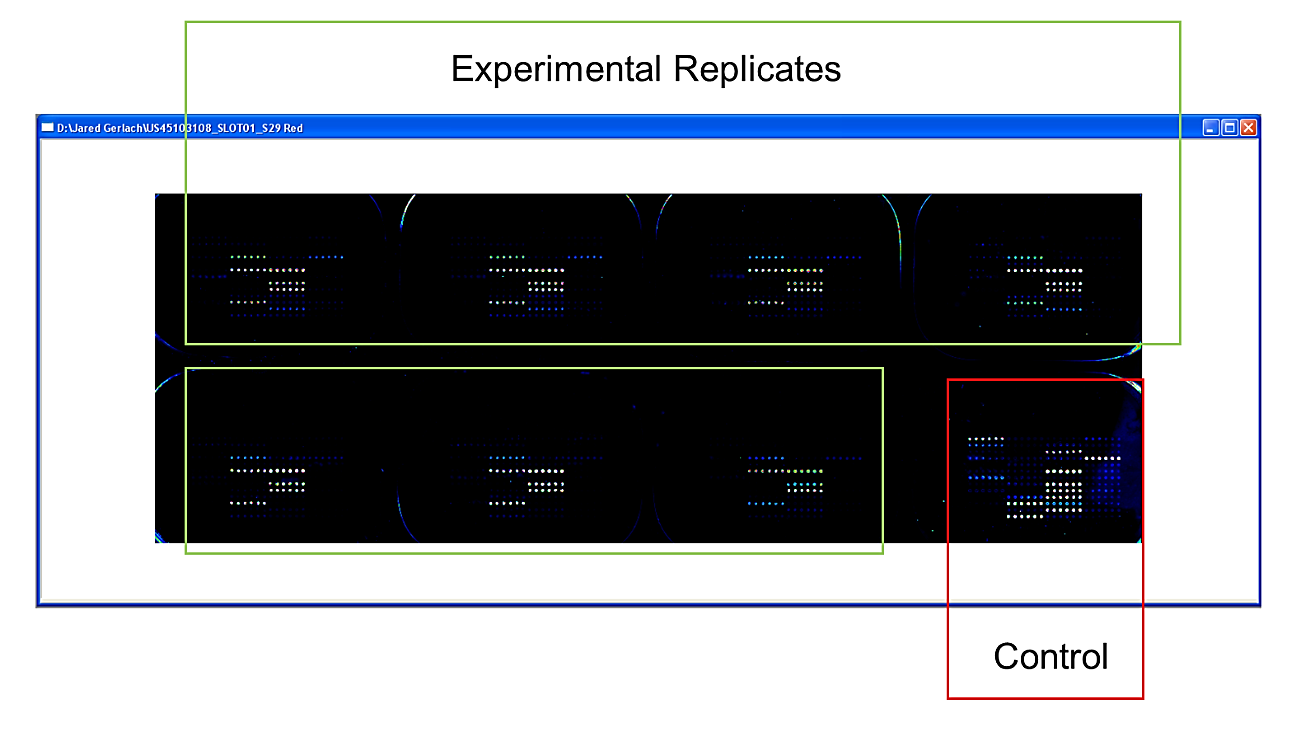


**Fig. S2.** Representative microarray image
